# Detecting Teeth Defects on Automotive Gears Using Deep Learning

**DOI:** 10.3390/s21248480

**Published:** 2021-12-19

**Authors:** Abdelrahman Allam, Medhat Moussa, Cole Tarry, Matthew Veres

**Affiliations:** School of Engineering, University of Guelph, Guelph, ON N1G 1W2, Canada; allama@uoguelph.ca (A.A.); ctarry@uoguelph.ca (C.T.); mveres@uoguelph.ca (M.V.)

**Keywords:** automated inspection, automotive gears inspection, gear defect detection, machine vision inspection

## Abstract

Gears are a vital component in many complex mechanical systems. In automotive systems, and in particular vehicle transmissions, we rely on them to function properly on different types of challenging environments and conditions. However, when a gear is manufactured with a defect, the gear’s integrity can become compromised and lead to catastrophic failure. The current inspection process used by an automotive gear manufacturer in Guelph, Ontario, requires human operators to visually inspect all gear produced. Yet, due to the quantity of gears manufactured, the diverse array of defects that can arise, the time requirements for inspection, and the reliance on the operator’s inspection ability, the system suffers from poor scalability, and defects can be missed during inspection. In this work, we propose a machine vision system for automating the inspection process for gears with damaged teeth defects. The implemented inspection system uses a faster R-CNN network to identify the defects, and combines domain knowledge to reduce the manual inspection of non-defective gears by 66%.

## 1. Introduction

Gears play an important role in automotive transmission systems, where they are used to transfer power from the vehicles motor through to the various wheels. The types of gears used in these transmissions are typically mass-produced, with different kinds of gears used in different vehicles, and all manufactured using different dyes and tooling processes. Due to these conditions, it is possible for a manufactured gear to either directly or indirectly be produced with a defect(s). Further, while these defects are relatively rare, due to the importance of the gear within automotive transmission, the defects must be caught before further downstream use and system assembly.

At an automotive gear manufacturer located in Guelph, Ontario, the current quality control inspection process requires human operators to manually inspect all gear for the presence of defects. However, the accuracy of such a system depends on the ability of the inspector to recognize defects in the gears. As the defects are infrequent and have diverse profiles, and as the gears themselves have different shapes and material characteristics (e.g., reflective surfaces), inspection can be a challenging and time-consuming process. All of these factors contribute to the quantity of parts that can be inspected daily, and as such, an automated system that can improve the inspection accuracy and speed is required to further optimize the quality control process.

### 1.1. Manufactured Gear Profiles

Four different configurations of helical gears are manufactured by this facility for use in automotive power transmissions. These gears have the same overall shape, but differ in their corresponding tooth profiles. The four gears share the same total length of 96 mm, a tooth length of 54 mm, and the same outer end shaft diameters (i.e., both shaft ends) of 40 mm and 50 mm, respectively. In addition, they have the same tapered inner profile, with the profile diameter increasing from 25 mm (inner) to 28 mm (outer). Table 1 presents the characteristics of the four different types of gears, as stated in the seminal work by Hall [1].

### 1.2. Manufactured Gear Defect Types

There are many different types of defects that can be observed on any of the manufactured gears [1]. These defects occur randomly on different gears, with no specific defects occurring for any specific gear type. In this work, we focus on detecting one of the most frequently observed defects, which is known as damaged teeth.

#### Damaged Teeth

**Figure 1:**
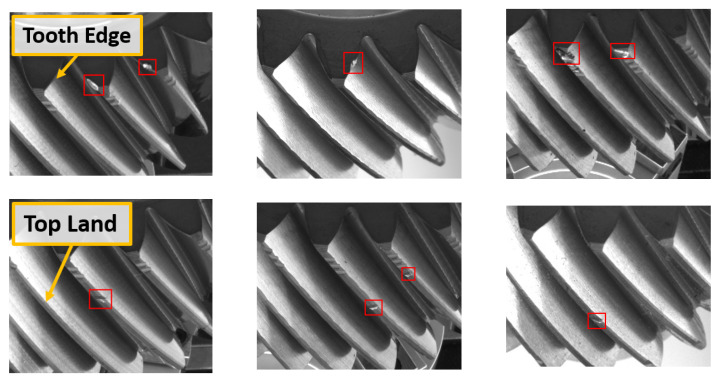
Damaged Teeth defect on the Tooth Edge (**top** row), and Top Land (**bottom** row).

The damaged teeth defect has a distinct profile on the gear, which usually appears as a laceration or lesion on the surface, and can be recognized by human operators through visual inspection. This defect can occur in different spots on a gear tooth, and can also appear on one or more teeth of the same gear. The most common sites where this defect is found includes the edge of a tooth and along the top land (the topmost surface of the tooth). When a defect is present on the edge of a gear tooth, the defect can cause a misalignment between the gears in the gearbox, which can in turn cause damage to the gearbox [2]. Figure 1 presents an example of the damaged teeth defect occurring on a gear “tooth edge” (top row), and on the “top land” (bottom row).

### 1.3. Contributions

In this work, we present a novel detection method for detecting damaged teeth defects that occur on automotive gears. The method is based on using a deep learning framework [3] combined with domain knowledge of a gear scan to provide a 100% scan accuracy for all gears. It is expected that this method will be used in combination with human inspection to improve the quality control process for manufactured automotive gears.

## 2. Literature Review

Machine learning techniques have a history of being used for manufactured part inspection [4,5,6]. However, within the past few years, progress in advanced machine learning techniques belonging to the field of deep learning, and the transition to an Industry 4.0 enabled environment, has encouraged the development of smarter and more intelligent inspection protocols.

With respect to vision-based tasks (e.g., such as defect inspection, in this work), one of the most promising families of deep learning models is known as convolutional neural network (CNN) [7]. Part of what has enabled this success is captured in the inductive spatial bias of the network, along with the fact that these networks can learn features of the data required to solve a task—rather than requiring humans to manually design them in advance of system deployment. For a complex task, such as gear defect detection, where defects and gears can have visually diverse and challenging profiles, deep learning is well positioned to tackle this problem.

Work by Zhou et al. [8] proposed a CNN model capable of learning both low level features (e.g., edges and corners) and high level features (e.g., objects) to classify surface defects on hot-rolled steel sheets. Extracting both feature levels enhanced the feature representation and, consequently, the performance of the classification. In [9], Song et al. proposed an inspection method based on CNN to classify four types of defects that appear on metal screws. The model was trained on a dataset of metal screws collected by the authors and scored an accuracy of 98%, which showed better results when compared to LeNet-5 [10] and traditional template matching methods.

In [11], Wen et al. proposed an algorithm to inspect the circularity of bearing rollers, as well as the defects that occurred on them. The Hough transform method [12] was used to detect the circular contour of the bearing and check for circularity, and a CNN was used to extract features of the bearing rollers to classify and locate four common defects.

While deep learning models typically require a significant amount of positive and negative examples (or, e.g., defective and non-defective labels) for training, the nature of manufacturing tends to shy away from this trend—producing parts that conform to some pre-defined specs the majority of the time, and only producing defective parts intermittently. Studies have applied different methods to overcome this problem, such as in [13], where Yun et al. proposed a data augmentation method (based on a variational autoencoder) to increase the amount of training data for steel surface defect classification.

Transfer learning is another technique that has been applied to combat issues of the low-sample label regime. Transfer learning makes use of models trained on one domain, and then transfers the learned knowledge (i.e., learned features) to a secondary, target dataset. In the visual domain, ImageNet [14] is a common source dataset used due to its vast size and sample diversity. In [15], Natarajan et al. applied a pre-trained CNN from ImageNet to detect metal surface defects. To improve the feature extraction process of steel surface defects, He et al. [16] added a multilevel feature fusion network (MFN) to the feature maps extracted from a CNN trained with the NEU-DET defect classification dataset [17]. This MFN integrates various hierarchical features into one feature, which contains more information about the location of the defects. A region proposal network (RPN) was included in this algorithm to generate regions of interest (ROIs). A detector was then applied to classify and locate defects on these ROIs. Other studies have applied both data augmentation and transfer learning. Zeng et al. [18] applied both methods to detect defects on a steel sheet, while Neuhauser [19] applied these methods to inspect the profiles of extruded aluminum.

## 3. Description of the Method

To detect the damaged teeth defect on gears, we propose a novel method that is based on integrating domain knowledge with the faster-RCNN deep learning model [20] trained using bounding-box annotations of the defect. The output of faster-RCNN includes the (1) bounding box proposals of areas in an image that look like the defect, and (2) the corresponding probability that the area actually contains the given defect. Below, we briefly discuss the structure of faster-RCNN [20], and then explore how predictions from this model can be incorporated in an automated inspection system to flag gears with defects. In particular, we focus on how domain knowledge can be applied to reduce the false-positive detection rate.

### 3.1. Defect Detection via Faster R-CNN

**Figure 2:**
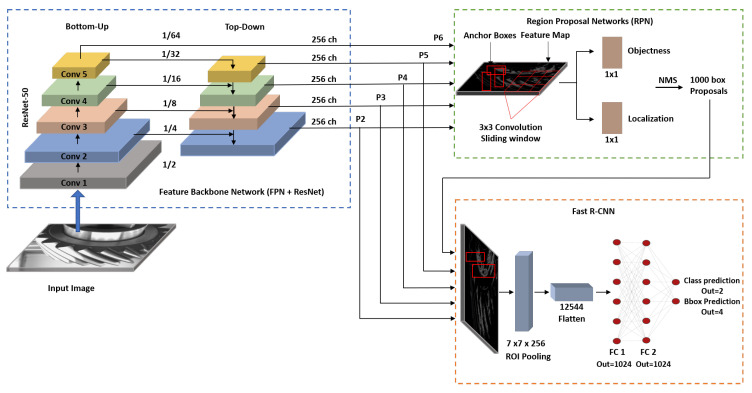
Faster R-CNN deep learning network for defect detection.

Faster-RCNN [20] is a CNN-based model that takes an image (e.g., RGB) as input, and proposes bounding-box localization(s) of target object(s) and corresponding class probabilities. Faster R-CNN (Figure 2) consists of three different network components:

1. A backbone network for feature extraction;
2. A region proposal network (RPN) [20] that identifies interesting areas of an image;
3. A fast R-CNN [21] for object classification and bounding box regression.

Given an input image (in this work we consider RGB images), the backbone network is used to extract features from the image that can be used for both object localization and classification further down the network pipeline. These features take the form of spatial (Width×Height×Depth) feature maps (depth = 256 channels is kept constant across the extracted layers), which are produced at different scales, depending on the current layer of the network. The backbone network combines both a feature pyramid network (FPN) [22] (with bottom-up and top-down pathways), and a residual neural network (ResNet) [23] with 50 layers.

The region proposal network takes the features maps from the backbone network, and slides a 3×3 convolutional filter over them to output a number of bounding boxes along with their probability scores of being an object (i.e., here, being a damaged teeth defect). At each sliding window position, anchors are generated with different scales and aspect ratios, to be considered as either a foreground or a background class. To have a balanced dataset, the network randomly selects labels from the background class and reduces the number of its labels to be equal to the foreground class.

Finally, the Fast R-CNN component takes both the output feature maps from the FPN and the predicted objects from the RPN, and performs both bounding box classification and bounding box regression. The network classifies the predicted object as being either a damaged teeth defect or a background class, and also outputs the four coordinates of the bounding box that contains the object.

### 3.2. Applying Domain Knowledge to Reduce False Positive Detections

Using the combined bounding box and class probability predictions from faster-RCNN (Section 3.1), a naïve gear inspection method could be designed to flag gear for further review if, e.g., a certain number of defect detections are made, or a certain classification threshold is reached. We should note that these predictions are made over individual images of the gear. Regardless of how confident the network may be in its predictions, such an approach could have a propensity for detecting false-positives, as, e.g., surface reflectance, dust, or defect profiles remain high. In turn, as more false-positives are predicted, a larger number of gears would be flagged for further manual review, and negatively impact the performance of the system.

**Figure 3:**
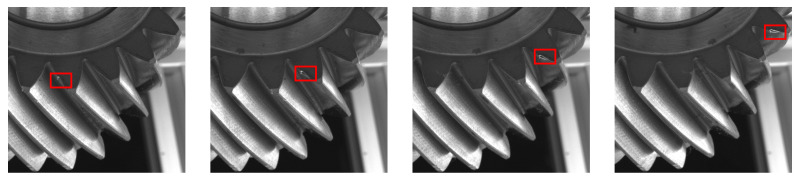
The same damaged teeth defect remains visible to the camera as the gear is rotated during inspection starting from the left most image to the right most image.

Instead we propose incorporating domain knowledge about the inspection process. We require the defect to be detected across multiple, sequential images of the gear scan. If a defect is detected on one tooth of the gear, the same defect should still remain visible if the gear is rotated slightly, albeit in a slightly different location. Figure 3 highlights this observation using four consecutive images with the same defect.

By combining the defect prediction probability (e.g., 75%, 80%, 85%) with the number of sequential images, we expect to see the defect in (e.g., 1, 2, or 3 sequential images), we can devise a heuristic that allows us calibrate the inspection system to reduce the number of false positive, gear-level predictions made by the system. We explore this setting further in Section 5.3.

## 4. Data Collection and Experimental Setup

Data were collected at the facility in Guelph, Ontario, over a period of several months. Quality control inspectors first identified gears that had one or more defects, and then set them aside for our team to scan and label further using a specially designed data acquisition system. The inspectors also set aside random gears without any defect.

### 4.1. Data Acquisition System

**Figure 4:**
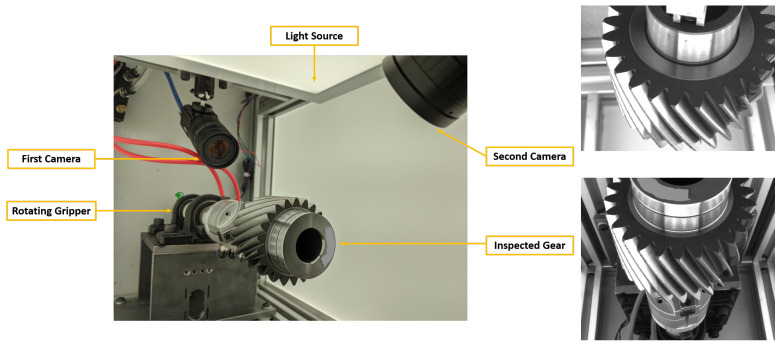
Inspection cell and sample camera images. **Left**: inspection cell, **Top Right**: image from the first camera, **Bottom Right**: image from the second camera.

Hall [1] and Cole Tarry developed a visual data acquisition system that could capture images of defective and non-defective gears inspected at this manufacturing facility. The system is equipped with two cameras installed at opposite ends of the inspection chamber, which together are able to capture the full extent of the gear to be inspected. A rotating gripper within the cell is used to hold the gear and rotate it such that every gear tooth can be imaged. We refer to the process of imaging every gear tooth as a full “gear scan”. Figure 4 shows the basic gear scanning and data collection setup.

The smallest defect targeted by the system has a size of 0.42 mm. The spatial resolution of the two cameras was previously calculated to be 2048×1536 and 2448×2048 pixels for the first and second cameras, respectively, corresponding to a size of 0.05 mm/pixel. In addition, light panels were installed inside the chamber to ensure that the defects could be seen.

### 4.2. Gear Scanning and Ground Truthing Procedure

As mentioned, every gear was first manually inspected by quality control personnel at the manufacturing facility to ensure label correctness. The inspector indicated on the gear whether a defect was present, and if so, the type of defect and general area it could be found. Information on the type of gear (Table 1) was also recorded prior to the gear scan.

After this information was recorded, the inspected gear was mounted on the rotating gripper, and the two cameras collected an image of the top-most gear tooth. The images were saved in a database, and then the gripper rotated such that the next tooth was aligned with the cameras. This process repeated for all 22 or 26 teeth before the system reset back to the start, and the next gear was scanned. We generally observed the scanning procedure to take around 90 s per gear, though note that the current system has not been optimized for speed, which is left for future work. We expect that optimizing the computing hardware and using continuous scanning will cut this time to around 20 s per gear.

A total of 193 gears with a damaged teeth defect, and 100 gears without a defect, were scanned by the system. All scanned gears were picked randomly from the manufacturing process without any bias to a specific outcome. The COCO annotator [24] was used to generate bounding-boxes around the scanned damaged teeth defects. From these scans, 1711 images were found to have a defect, and a total of 3172 defects were labeled. The labeled defects had sizes ranging from 0.55 to 10.45 mm, and an average of 2.04 mm. All sizes were measured using the largest side of the defect. During the data collection process, we did not notice any instances where multiple types of defects were present on the gear at the same time.

### 4.3. Training Parameters

Our faster-RCNN model was built using Facebook’s Detectron2 python library [3]. For training the network, we considered only one foreground class (i.e., damaged teeth defects), and used a batch size of four images. We fine-tuned a faster R-CNN using a pre-trained ResNet-50 backbone, and SGD optimizer. We used a learning rate of 0.01, and trained the network for 10,000 iterations.

## 5. Evaluation

To evaluate our model, we tested how well it could (1) detect defects on images; (2) detect defects on different gears; and (3) flag gears for further, manual inspection in an industrial setting.

### 5.1. Cross Validation on Images with Defects

**Figure 5:**
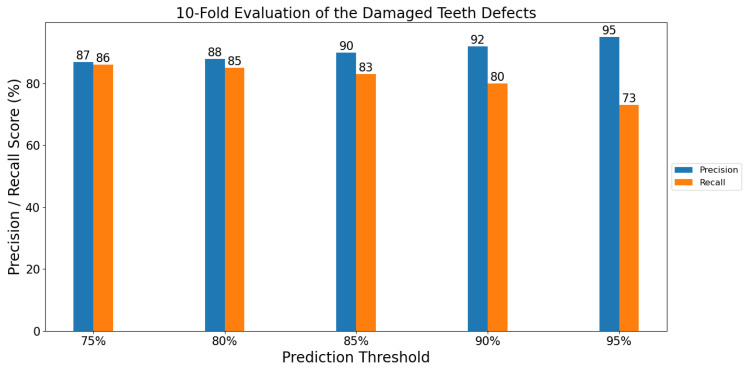
Average precision and recall of the 10-folds for the damaged teeth defects.

To evaluate the general ability of our model to detect the damaged teeth defect, we trained the model using a 10-fold cross validation strategy and the dataset of 1711 defect images. In each fold, 90% of the images were used for training and 10% of the images were used for testing. Figure 5 shows the average precision and recall results.

As shown in Figure 5, the model performed well in terms of both precision and recall among the various confidence thresholds. When a classification threshold of 75% was used (i.e., at ≥75% probability of being a damaged tooth defect), the model was able to detect 86% of the defects (recall) and maintain a precision of 87%. As the prediction threshold was increased, the model began to miss some defects (lower recall), but also began to eliminate more false positives.

### 5.2. Evaluation on Images with Defects on 30 Gears

**Figure 6:**
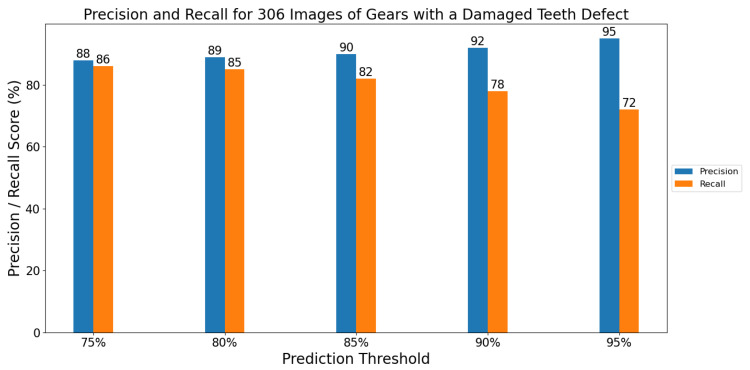
Precision and recall values for 306 images of damaged teeth defects.

Thirty (30) gears were randomly selected from the set of 193 gears to form an independent test set to evaluate our model gear inspection performance. We note that this test set was preserved only for testing the model and it was not included in any stages of training or validation of the model. In this test set, there are 306 images with damaged teeth defects, and a total of 554 defects labeled. The remaining 163 gears were used as a training set, and included 1405 images with a defect. Figure 6 shows the model precision and recall performance on the test, after re-training on the data split. When a prediction threshold of 75% was chosen, the model achieved a precision and recall score of 88% and 86%, respectively. The model also showed good precision and recall percentages when the prediction score was increased.

### 5.3. Whole Gear Inspection and Industrial Validation

The evaluations in Section 5.1 and Section 5.2 were performed on individual images of gears that had damaged teeth defects, and was used to evaluate how well the faster-RCNN model was able to detect them. However, in an industrial setting, using only the prediction confidence and detection for a single image could possibly send a large number of gears for manual inspection due to the potential for detecting false-positives. In this experiment, we applied our faster-RCNN model (Section 5.2) to the entire gear scan of both defective and non-defective gears, and evaluated the model performance on identifying defective gears when the consecutive image constraint was applied.

First, we evaluated our model (faster R-CNN and the consecutive image constraints) on gears *without* a defect, to understand how many gears would be detected as a false positive. Table 2 shows the results of our model when applied to the set of 100 defect-free gears collected in Section 4.2. Note that as these gears were manufactured without a defect, any predictions satisfying these constrains would correspond to a “defect-free” gear that would have to undergo further, manual human inspection.

**Figure 7:**
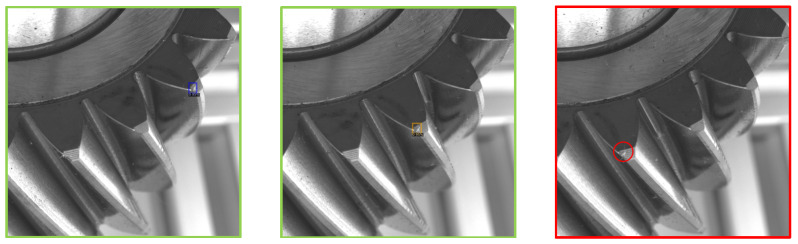
A false positive was not considered a defect since it was detected by the algorithm only on the first and second images (surrounded by blue and yellow bounding boxes), and was not detected on the third image (red circle).

From Table 2, it can be seen that, as the number of consecutive image requirements increased, the number of gears that were flagged as having a defect would decrease accordingly. Compared to the case when only one image was used, the results show that using the multiple consecutive image constraints resulted in a significant decrease in false positives reaching almost 50% reduction with three images and 66% reduction with four images. Intuitively, this means that for gears without a defect, the model would have to make one or more false-positive detections across multiple sequential images, in order for the gear to be flagged as having a defect. This could be seen from Figure 7, where an example of a false positive was not picked as a defect when a threshold of three consecutive images and a prediction confidence of 90% were applied. Thus, as long as the system has an ability to detect defects on a gear (e.g., Figure 6), we can further improve the false-positive detection performance by requiring defects to be predicted across multiple, sequential images of the gear. We do not present results beyond four consecutive images due to two factors. Some defects (which appear on the “top land” of the tooth) will not be visible unless they are close to the centre of the image. Moreover, lenses suffer from optical distortions away from the optical center.

When tightening the constraints on the number of consecutive images and prediction confidence, from Figure 6, we expect the model to also miss detecting some gears with a defect. That is, because we require predictions with strong confidence to be made across multiple images, if a gear has a defect, but these requirements are not met, then the system would fail to flag the gear for manual inspection. Table 3 presents the false-negative prediction results, when applying our model and constraints on the test set of 30 defective gears (Section 5.2).

Table 3 indicates that, when we set a classification threshold of ≥90% and required a defect to be detected in three images, the model was able to successfully detect 100% of the defective gears (i.e., 0% false negatives). When the number of consecutive images was set higher however (e.g., requiring four images to have a predicted defect), some of the defective gears were missed by the model. Together, Table 2 and Table 3 suggest that, at a consecutive image constraint of images = 3, and a confidence threshold of ≥90%, the model would be able to detect 100% of the gears with a defect (Table 3), while also reducing the manual inspection of the non-defective gears to 34% (Table 2).

## 6. Discussion and Conclusions

The proposed deep learning model for damaged teeth defect inspection performed well when the domain knowledge constraint was added. The best results came about by setting the prediction threshold to 90% and the number of consecutive defective images that needed to contain a defect to three images. In addition, only 34 of the 100 non-defective gears were classified as defective. This result indicates that, rather than manually inspecting all 100 gears, the number of gears requiring manual inspection could be reduced by a total of 66%. Adding the domain knowledge constraint of requiring sequential images to contain a defect adds only minimal complexity to the inspection systems.

In this work, we focused on training a single model to detect a single defect. When applied in a real-world industrial setting, there are several benefits to this kind of approach: from the obvious ability allowing a model to specialize to detect a single type of defect (damaged teeth defect), to the lesser obvious, but still important aspects of model maintenance. Adding a new type of defect to the detection pipeline would require a new model to be trained—rather then performing the time-consuming and laborious process of re-updating the current model, again, for every defect. In addition, credit assignment and blame becomes more straightforward for management personnel in these situations.

With these considerations in mind, in future work, we seek to capture more defects that can occur on the gears, and evaluate the performance of a single unified defect detection model. We also plan to evaluate the model performance when more labeled data are available.

## Figures and Tables

**Table 1:** Characteristics of the four types of gears produced by the factory in Guelph, Ontario [1]. Diameters and angles are in mm and degrees, respectively.

Gear Type	Teeth	Pitch Diameter	Helix Angle	Pressure Angle	Major Diameter	Minor Diameter
A	22	69.57	23.5	22.5	78.5	62
B	22	66.437	22.5	22.5	75.2	59.5
C	26	73.368	21.25	20	82	66.5
D	22	63.953	21.75	22.5	72.4	57.5

**Table 2:** False positive percentages when testing the system on 100 non-defective gear scans. “Images = X” represents the number of sequential images of the gear scan where a defect was predicted with a probably greater than the corresponding prediction confidence.

Prediction Confidence	Images = 1	Images = 2	Images = 3	Images = 4
75%	94%	69%	49%	34%
80%	91%	60%	44%	32%
85%	83%	57%	40%	28%
90%	78%	48%	34%	24%
95%	57%	36%	24%	14%

**Table 3:** False negative percentages with consecutive image constraints on 30 defective-gear scans. “Images = X” represents the number of sequential images of the gear scan where a defect was predicted, probably greater than the corresponding prediction confidence.

Prediction Confidence	Images = 1	Images = 2	Images = 3	Images = 4
75%	0	0	0	6.70%
80%	0	0	0	10%
85%	0	0	0	10%
90%	0	0	0	13.30%
95%	0	0	3.30%	23.30%

## Data Availability

Restrictions apply to the availability of data used in this paper. Data was obtained from an industrial partner and are available from the authors with the permission of the industrial partner.

## References

[1] Hall G. (2020). A Data Collection System for Future Automation Applications. Master’s Thesis.

[2] Panwar V., Mogal S. (2015). A Case Study on Various Defects Found in a Gear System. Int. Res. J. Eng. Technol..

[3] Wu Y., Kirillov A., Massa F., Lo W.Y., Girshick R. Detectron2. https://github.com/facebookresearch/detectron2.

[4] Shi T., Kong J.Y., Wang X.D., Liu Z., Zheng G. (2016). Improved Sobel algorithm for defect detection of rail surfaces with enhanced efficiency and accuracy. J. Cent. South Univ..

[5] Wang Y., Xia H., Yuan X., Li L., Sun B. (2018). Distributed defect recognition on steel surfaces using an improved random forest algorithm with optimal multi-feature-set fusion. Multimed. Tools Appl..

[6] Wu G., Zhang H., Sun X., Xu J., Xu K. A bran-new feature extraction method and its application to surface defect recognition of hot rolled strips. Proceedings of the 2007 IEEE International Conference on Automation and Logistics.

[7] LeCun Y., Bengio Y., Hinton G. (2015). Deep learning. Nature.

[8] Zhou S., Chen Y., Zhang D., Xie J., Zhou Y. (2017). Classification of Surface Defects on Steel Sheet Using Convolutional Neural Networks. Mater. Technol..

[9] Song L., Li X., Yang Y., Zhu X., Guo Q., Yang H. (2018). Detection of Micro-Defects on Metal Screw Surfaces Based on Deep Convolutional Neural Networks. Sensors.

[10] Lecun Y., Bottou L., Bengio Y., Haffner P. (1998). Gradient-based learning applied to document recognition. IEEE.

[11] Wen S., Chen Z., Li C. (2018). Vision-Based Surface Inspection System for Bearing Rollers Using Convolutional Neural Networks. Appl. Sci..

[12] Hough P.V. (1962). Method and Means for Recognizing Complex Patterns. U.S. Patent.

[13] Yun J.P., Shin W.C., Koo G., Kim M.S., Lee C., Lee S.J. (2020). Automated Defect Inspection System for Metal Surfaces Based on Deep Learning and Data Augmentation. J. Manuf. Syst..

[14] Deng J., Dong W., Socher R., Li L.J., Kai L., Fei-Fei L. ImageNet: A Large-scale Hierarchical Image Database. Proceedings of the 2009 IEEE Conference on Computer Vision and Pattern Recognition.

[15] Natarajan V., Hung T.Y., Vaikundam S., Chia L.T. Convolutional Networks for Voting-based Anomaly Classification in Metal Surface Inspection. Proceedings of the 2017 IEEE International Conference on Industrial Technology (ICIT).

[16] He Y., Song K., Meng Q., Yan Y. (2020). An End-to-End Steel Surface Defect Detection Approach via Fusing Multiple Hierarchical Features. IEEE.

[17] Song K., Yan Y. (2013). A noise robust method based on completed local binary patterns for hot-rolled steel strip surface defects. Appl. Surf. Sci..

[18] Zeng W., You Z., Huang M., Kong Z., Yu Y., Le X. Steel Sheet Defect Detection Based on Deep Learning Method. Proceedings of the 2019 Tenth International Conference on Intelligent Control and Information Processing (ICICIP).

[19] Neuhauser F.M., Bachmann G., Hora P. (2020). Surface Defect Classification and Detection on Extruded Aluminum Profiles Using Convolutional Neural Networks. Int. J. Mater. Form..

[20] Ren S., He K., Girshick R., Sun J. (2016). Faster R-CNN: Towards Real-Time Object Detection with Region Proposal Networks. arXiv.

[21] Girshick R. (2015). Fast R-CNN. arXiv.

[22] Lin T.Y., Dollár P., Girshick R., He K., Hariharan B., Belongie S. (2017). Feature Pyramid Networks for Object Detection. arXiv.

[23] He K., Zhang X., Ren S., Sun J. Deep Residual Learning for Image Recognition. Proceedings of the 2016 IEEE Conference on Computer Vision and Pattern Recognition (CVPR).

[24] Brooks J. COCO Annotator. https://github.com/jsbroks/coco-annotator.

